# Service as joint editor-in-chief for 11 years comes to an end: adieu, godspeed and auf wiedersehn!

**DOI:** 10.1186/s13049-015-0192-1

**Published:** 2015-12-30

**Authors:** Kjetil Søreide

**Affiliations:** Department of Gastrointestinal Surgery, Stavanger University Hospital, PO Box 8100, Stavanger, Norway; Department of Clinical Medicine, University of Bergen, Bergen, Norway

Bidding farewell and breaking up from a good company is never easy, yet every table setting has its end. For me, after serving an 11-years term as joint Editor-in-Chief (2005–2015), it is now time to look back at a remarkable journey before I say ‘adieu’. Indeed, a journey that turned out more fabulous than anyone would have believed or maybe even dreamed of at the time of its onset. My inaugural article in the Journal [1] focused on how to read a paper and, at the time, SJTREM was not an obvious choice among the established journals in emergency medicine. Since then, SJTREM has firmly placed itself as a steady growing, serious player and solid vehicle of scientific work in Scandinavia and beyond. A retrospective view is thus timely and should point to a still prosperous future growth for the Journal.

First and foremost, I have to sincerely thank my co-Editor-in-Chief, professor Hans Morten Lossius for putting his trust in me at a very early stage in my career. Also, the persistence and collaboration with deputy editor over many years, Mr. Kjetil G. Ringdal MD, PhD has been very much appreciated. Knowingly, so many people have been involved over the several years, so ‘none mentioned, none forgotten’, but all efforts have been very much appreciated, up until the current composition of the editoral board (www.sjtrem.com).

In a time of transition from what was then a local/national magazine under the name “Akuttjournalen” based on opinion pieces, reports and organizational material of interest primarily to the national prehospital community in Norway, the desire to become a more international based scientific and academic journal based in Scandinavia was launched. The journal idea was among others an offspring of many successful international conferences, including the Traumacare2002 conference [2] that coined the chain of survival terminology (Fig. 1), followed by other conferences such as Scandinavian Updates in 2005 [3] and 2009 [4] and Resuscitation 2006 (Fig. 2) to mention but a few.

**Fig. 1:**
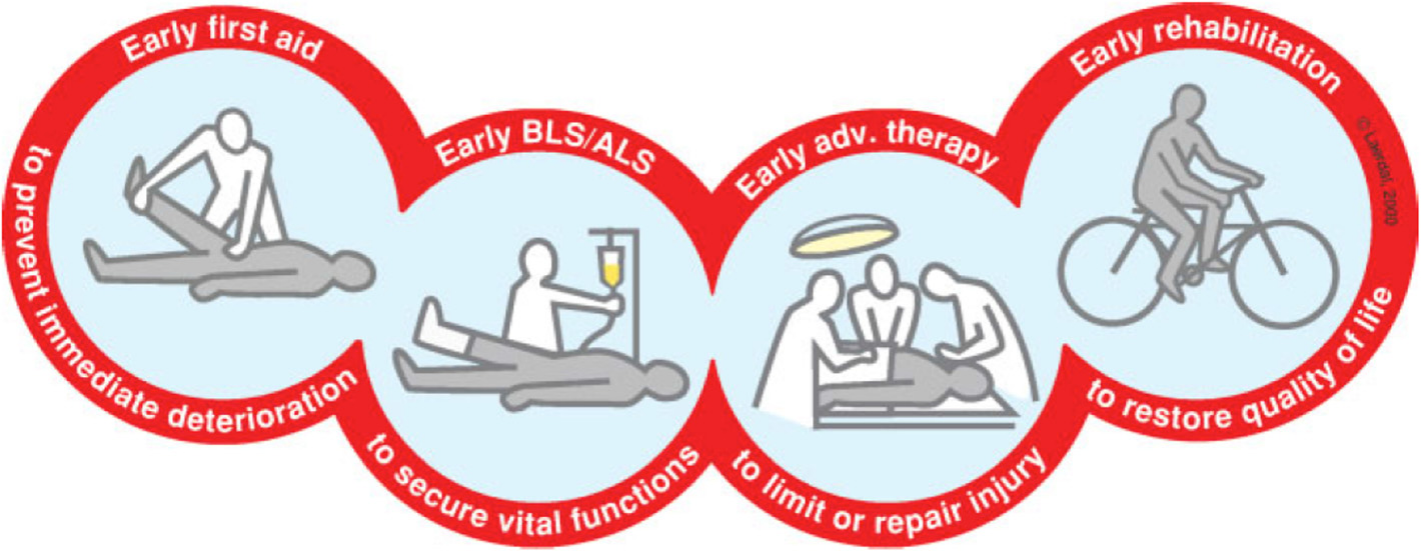
The trauma chain of survival. Reproduced from Laerdal Medical, ©Lærdal 2000

**Fig. 2:**
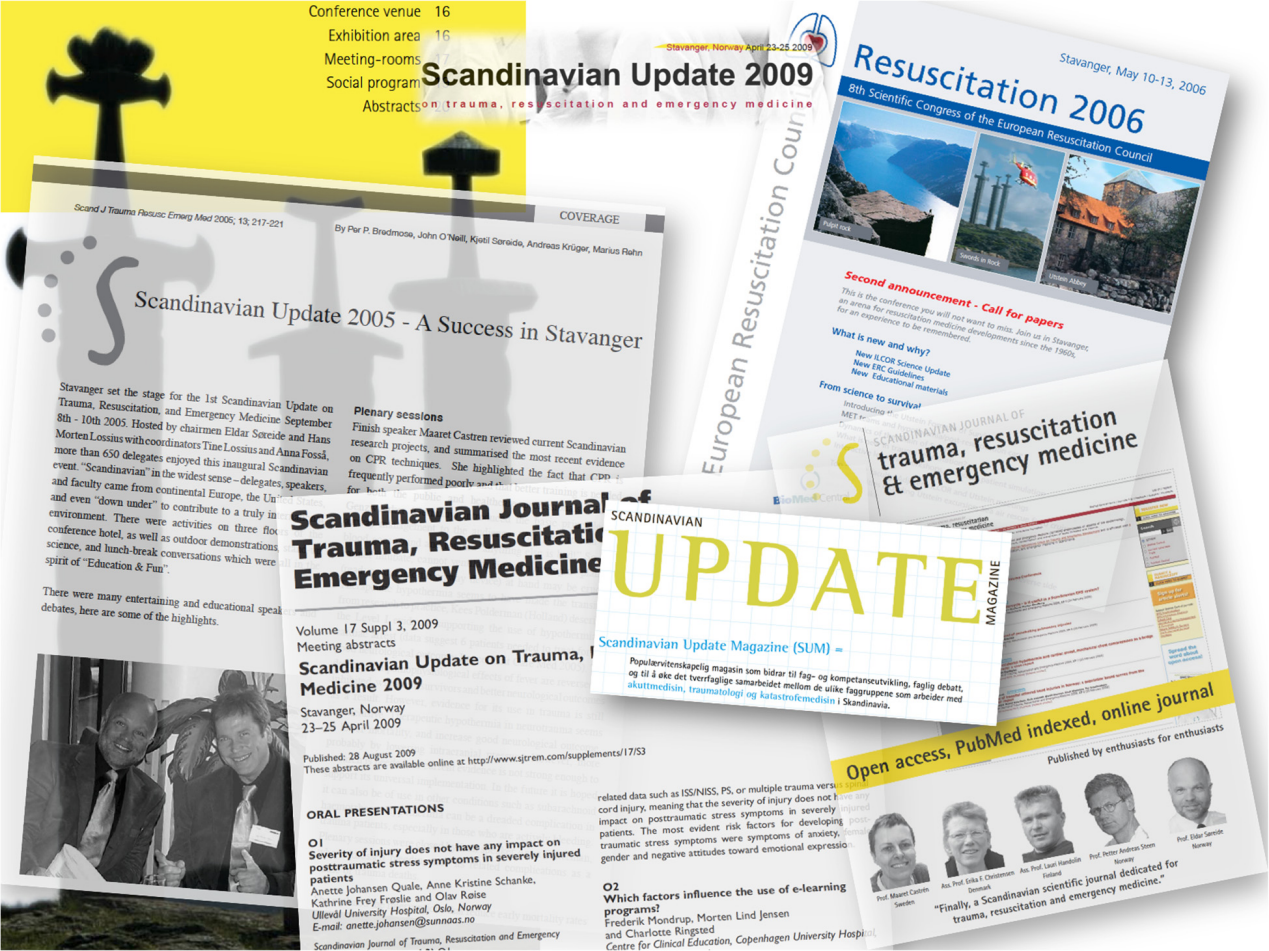
The formative years of the early journal development. Snapshots from some of the conferences and meetings

Several ‘hairy’ goals were launched at an editorial meeting in Copenhagen, such as becoming a strictly peer-reviewed journal; focus on original and review papers; decision to cover aspects of multidisciplinary care throughout the chain of survival; focus on clinical research along the themes of trauma, resuscitation and emergency medicine; and, becoming the lead Scandinavian journal in its category. On top of this we were determined to be eligible for registration in the PubMed/Medline system, and, last but not least, achieve an impact factor by inclusion in the Thomson Reuters Journal Scientific Report. The latter one seemed a bit out of range in itself, but we determined not only to do this, but also with the goal of establishing an impact factor (IF) above 2.0.

Indeed, I believe many would regard this as a mouthful (and, yes, it was), but goal-directed team efforts spurred success. I firmly believe the initial strong support by several thought leaders in Scandinavia [5–9], Europe [10–13] and North America [14–21] was crucial – their submission of timely topics from expert groups and world-leading centres in an amalgam of evidence- and education-based material provided for several strong review articles across several disciplines [5–7, 10, 14–23], several of which continue to be accessed, downloaded and heavily cited in the Journal even today.

The name change (from Akuttjournalen) and transition was not easy, and among the several options, we landed on a name that is not easily spoken in one breath: the *Scandinavian Journal of Trauma, Resuscitation and Emergency Medicine* is one mouthful for sure! Maybe that is why many have taken to the abbreviated name – SJTREM – as their preferred usage. Tongue in cheek, some prefer it with a French twist on the pronunciation, dubbing ‘SJTREM’ into an almost French-like *j’taime,* with an ‘r’ [ʒ ə.tr‿ɛ m]. I guess this only testifies to the Journal eventually having gained the love and acclaim of the community – *j’taime SJTREM*!

The evolution of the Journal has been documented in the past [24–29], with the first PubMed indexed paper in 2008 [30], and the first impact factor (IF) obtained already in 2010, for a strong debut of 2.176! Breaking the 2.0-barrier at first attempt surely surprised the most hard-core of sceptics and certainly those who viewed this an ephemeral enterprise. However, after a dip in IF (2012 at 1.68) that coincided with a rapid increase in submission and accepted papers, the IF has gained momentum and is now over 2.0. With the continued increase in IF over the past 2 years (about 20 % increase, the IF could very well land at 2.4 and above in 2-years time. Notably, this is unpredictable and largely dependant on factors out of the hands of the editors. Also, IF is a debated metric for quality, influence and content relativity so it should not be viewed as a sole quality indicator or measure of importance. Indeed, the journal is at strength with others, and lists in the top 5 in its category, clearly scoring higher than many long-lasting and established competitor journals (Table 1). Knowing that IF is not the only but one way of viewing the quality or impact of any given journal, it is assuring to see that SJTREM has established itself as an alternative and competitive voice in its category. While lacking proof of the claim, I also hold the belief that it has inspired and driven more and better research in trauma, resuscitation and emergency medicine in Scandinavia.

**Table 1:** Top 20 journals in the “emergency medicine” category

Rank	Journal name (abbreviation)	IF^a^
1	Ann Emerg Med	4.695
2	Resuscitation	4.167
3	Emergencias	2.895
4	Injury	2.137
5	**Scand J Trauma Resusc Emerg Med**	2.025
6	Acad Emerg Med	2.006
7	Emerg Med J	1.843
8	Prehosp Emerg Care	1.763
9	Eur J Emerg med	1.583
10	World J Emerg Surg	1.473
11	Emerg Med Australas	1.296
12	Am J Emerg Med	1.274
13	Can J Emerg Med	1.163
14	Pediatr Emerg Care	1.046
15	J Emerg Med	0.969
16	J Emerg Nurs	0.787
17	Emerg Med Clin N Am	0.778
18	Unfallchirurg	0.649
19	Notfall Rettungsmed	0.472
20	Eur J Trauma Emerg Surg	0.346

^a^based on 2014 Web of Science data

Thus, it is with much pride and few concerns that I shall demit office after 11 exciting years. The Journal appears to be in sound shape, with good prospects and with a steady course towards the future. I am proud of having been part of its inception, evolution, development and eventual establishment as a Journal with a strong foothold in Scandinavia and beyond.

Knowing continuity will be assured with professor Hans Morten Lossius still involved, I welcome professor David Lockey from London HEMS to succeed as joint EiC of the Journal. The past managing editor Kristi G. Bache PhD now takes over as EiC and will continue to maintain the day-to-day contact with authors, referees and publishers alike. New faces and new places will be introduced only to the better advancement for the Journal. Still, I hope a Scandinavian focus will remain visible, although the world is now the playground for us all.

Last but not least, I sincerely need to thank the most important contributors to the success of the Journal. For one, the many named and unnamed referees who, relentlessly and with dedication, have put time, knowledge and expertise into reviewing the numerous papers submitted to the Journal over the years. Without your efforts and dedication, the Journal could not exist. Second, to the number of authors who have contributed original and review papers of very high quality at a time when the Journal was trying to get a foot in the door to the academic community of people interested in trauma, resuscitation and emergency medicine. Many high-ranked and high profiled authors devoted of their time and expertise to contribute timely pieces on their pet topics. For sure, this has largely contributed to the growth and development of the journal. Finally, my sincere gratitude and expressed thanks goes to those who peruse the Journal material, be it as readers of the content, presenters at conferences and as citations in further publications. The steady and increasing use of SJTREM material in other media and in meetings testifies to its having a role and being a vehicle for transmission of knowledge across Europe and beyond. Lastly, the ultimate goal of the Journal and its content is to advance the medical care for the benefit of critically ill patients around the world.

So, it is only appropriate that I shall say not only ‘*takk for meg*’ but also include an ‘*adieu*’, forward a sincere wish of ‘*godspeed*’, and, definitely state an “*auf wiedersehn*” to the global supporters of the Journal.

## Abbreviations

IF: impact factor
SJTREM: Scandinavian Journal of Trauma Resuscitation and Emergency Medicine
HEMS: helicopte emergency medicine system
EiC: editor-in-chief
